# Nocardia Isolation in People with Cystic Fibrosis and Non-CF Bronchiectasis: A Multicenter Italian Study

**DOI:** 10.3390/antibiotics14030317

**Published:** 2025-03-18

**Authors:** Laura Venditto, Daniela Dolce, Silvia Campana, Pamela Vitullo, Marco Di Maurizio, Cristina Fevola, Francesca Lucca, Giovanni Taccetti, Vito Terlizzi

**Affiliations:** 1 Cystic Fibrosis Center, Azienda Ospedaliera Universitaria Integrata, 37126 Verona, Italy; 2 Department of Paediatric Medicine, Cystic Fibrosis Regional Reference Centre, Meyer Children’s Hospital IRCCS, 50139 Florence, Italy; 3 Cystic Fibrosis Support Center, Ospedale G. Tatarella di Cerignola, 71042 Cerignola, Italy; 4 Department of Radiology, Meyer Children’s Hospital IRCCS, 50139 Florence, Italy

**Keywords:** bronchiectasis, cystic fibrosis, primary ciliary dyskinesia, nocardia, antibiotics

## Abstract

**Background:** *Nocardia* species are an emergent pathogen in people with CF (pwCF) or bronchiectasis. Their clinical role and management remain unclear, and their isolation is a challenge. In this paper, we describe four cases of Nocardia detection, in two pwCF and two patients with non-CF bronchiectasis or primary ciliary dyskinesia (PCD). **Methods:** We conducted a multicenter retrospective study, involving pwCF and non-CF people with bronchiectasis who presented with a *Nocardia* detection and were followed at three CF Italian centers (Florence, Verona, and Cerignola). **Results:** *Nocardia* detection was associated with clinical and radiological respiratory exacerbation and decline in lung function. In one CF patient, *Nocardia* was not detected in sputum cultures after starting Elexacaftor-Tezacaftor-Ivacaftor therapy. **Conclusions:** Managing *Nocardia* detection in patients with underlying lung diseases such as CF, PCD, or bronchiectasis presents significant challenges for clinicians.

## 1. Introduction

*Nocardia* are ubiquitous Gram-positive bacilli with branching filamentous forms that can cause skin, lung, or brain infections, particularly in immunocompromised patients [1], or in patients with chronic obstructive pulmonary disease [2] and bronchiectasis [1].

Bronchiectasis is a chronic lung condition characterized by bronchial dilation resulting from a dysregulated inflammatory response that contributes to lung damage [3]. This creates a vicious cycle, first described by Cole et al. [4], where recurrent airway infections, inflammation, mucociliary dysfunction, and structural lung damage are the major drivers. Genetics and environmental factors may also play a role, although their precise contribution remains unclear [3]. In the presence of bronchiectasis, ciliary dysfunction, increased mucus production with altered viscoelastic properties, impaired local airway mucosal immunity, mucus plugging due to airway enlargement, and persistent airway inflammations can contribute to increased epithelial vulnerability to infections [3].

In recent years, there has been increased focus on the microbiology pattern of patients with bronchiectasis [5], with an increasing trend in *Nocardia* species detection in these patients [1]. This trend remains unclear, but may be linked to the increasing use of corticosteroids [1]. Furthermore, *Nocardia* infection itself may contribute to bronchiectasis pathogenesis, as pulmonary nocardiosis can present with a nodule-bronchiectasis pattern [6]. People with cystic fibrosis (pwCF) have a higher risk of nocardiosis, not only due to the presence of bronchiectasis, which creates a favorable environment, but also in cases of prolonged systemic steroid treatment, *Pseudomonas aeruginosa* (PA) colonization, and previous diagnosis of allergic bronchopulmonary aspergillosis [7]. In pwCF, *Nocardia* may act as a colonizer rather than a pathogen, but this remains debated in the literature [7]. This is particularly true regarding the criteria for treatment initiation and the optimal antibiotic regimen. Moreover, *Nocardia* may be underdiagnosed in pwCF, as sputum culture can fail to identify *Nocardia* compared to the novel next-generation sequencing (NGS) techniques [8], especially for pwCF in treatment with CFTR modulators, who may have difficulty expectorating, posing a challenge for clinicians [9]. Moreover, few cases of people with bronchiectasis and *Nocardia* detection have been described and there is no consensus on *Nocardia* treatment.

In order to increase the knowledge on this topic, in this study, we present the clinical presentation and management of *Nocardia* isolation in pwCF or patients with primary ciliary dyskinesia (PCD) or with bronchiectasis.

## 2. Results

Four patients with bronchiectasis had at least one *Nocardia* detection and were included in the study. In total, two patients were affected by CF and two patients with non-CF bronchiectasis. The median age at the time of the first *Nocardia* isolation was 26 (range of 18–33). Two patients were male. The median FEV_1_ was 78.5%pred (range of 61–94%pred). All the patients had a good BMI. Of the patients, 75% (*n* = 3) presented an acute exacerbation at the first *Nocardia* detection, with fever, cough, and increased mucus production. Two cases (50%) needed hospitalization and intravenous antibiotics. Of the patients, 75% presented with a relapse despite antibiotic therapy. After *Nocardia* treatment, the lung function improved or remain stable in the totality of the patients. Demographical data and clinical features are summarized in Table 1 (CF patients) and Table 2 (non-CF patients).

### 2.1. Case 1

We present the case of an adult man with CF (CFTR genotype: F508del/541DelC; sweat chloride (SC): 84–86 mmol/L) diagnosed at 4 months in the presence of pancreatic insufficiency and respiratory symptoms. He receives care at the CF center of Florence, Italy, according to the European Cystic Fibrosis Society (ECFS) standards of care [10]. During the follow-up, he developed lung disease with diffuse bronchiectasis at the computed tomography (CT) scan and experienced frequent pulmonary exacerbations. He also developed insulin-dependent diabetes mellitus, non-cirrhotic CF-related liver disease, and nasal polyposis, which was surgically treated. Sputum microbiology revealed chronic colonization by PA [11] from the age of 16 years, with intermittent colonization by methicillin-susceptible *Staphylococcus aureus* (MSSA), *Aspergillus fumigatus,* and the *Scedosporium apiospermum* complex.

**Figure 1:**
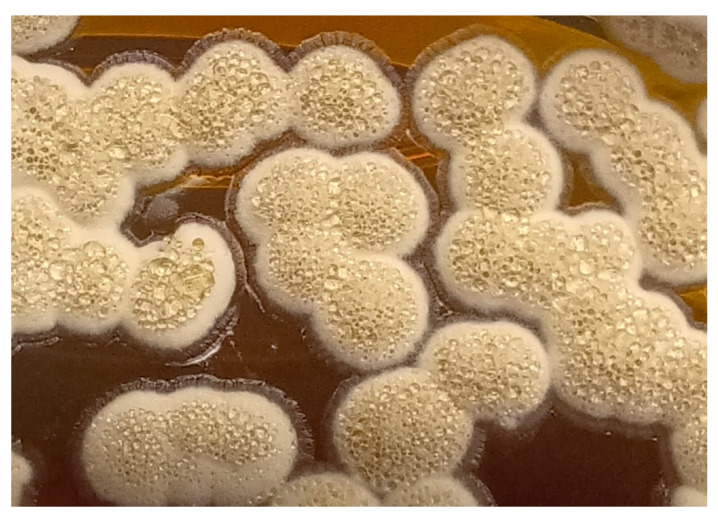
Nocardia macroscopic growth on nutrient agar medium.

From the age of 33 years, *Nocardia* species were isolated from sputum cultures (Figure 1). Clinical and diagnostic features of the different episodes of *Nocardia* isolation are reported in Table 1. At the first detection of *Nocardia*, in the absence of symptoms, new radiological findings, or worsening in lung functional tests, an eradication attempt was made with a one-month course of trimethoprim-sulfamethoxazole (TMP-SMX) according to its antimicrobial susceptibility. This eradication attempt was unsuccessful. Subsequent recurrences of *Nocardia* detection were observed, often accompanied by a substantial decrease in forced expiratory volume in the first second (FEV_1_). Some of these episodes were treated with oral home therapy (TMP-SMX and ciprofloxacin).

Following these antibiotic courses, he developed right basal pneumonia associated with pleural effusion. Given the chronic PA colonization, the patient was hospitalized for intravenous therapy with Ceftazidime and Tobramycin for 14 days, resulting in clinical improvement. At the age of 40 years, he initiated treatment with ETI therapy, according to the Italian legislative directives. This led to an improvement in FEV_1_ (from 61% to 79%), a reduction in cough and antibiotics use, and a normalization of the SC after one month (27 mmol/L). Since the initiation of ETI therapy in 2021, *Nocardia* has not been detected in the patient’s sputum cultures.

### 2.2. Case 2

We present the case of an adult woman diagnosed with CF at 12 years and 7 months (*CFTR* genotype: 3849 + 10 kbC > T)/3849 + 10 kbC > T, SC: 59 mmol/L) with pancreatic insufficiency, and recurrent chest infections. She receives care at the CF Center of Cerignola, Italy, according to ECFS standards of care [10]. According to the Italian legislative directives, it was not possible to prescribe ETI in the absence of at least one F508del variant.

Chest CT scan revealed diffused cylindrical bronchiectasis, which was more pronounced in the bilateral upper lobes. Endoluminal mucous plugs were observed in the subsegmental branches afferent to the anterior segment of the left lower lobe. She experienced approximately three pulmonary exacerbations per year, requiring intravenous antibiotic treatments, while maintaining good lung function (mean FEV_1_ of 98%).

**Figure 2:**
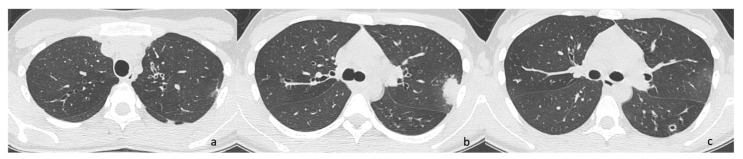
(**a**) Bronchiectasis with wall thickening in the left upper lobe. (**b**) Parenchymal pneumonia in the apical-posterior segment of the left upper lobe with pleural reaction. Concomitant peripheral ground glass. (**c**) Cavitated parenchymal lesion with thickened walls.

Sputum microbiology analysis has identified persistent colonization by MSSA and PA since the age of 13 years. At the age of 26 years, she presented with an acute respiratory exacerbation characterized by chest pain, fever, increased cough, minor hemoptysis, a decline in FEV_1_ from 98% to 84%, and left apical pneumonia at chest X-ray. Chest CT scan confirmed consolidation in the left upper lobe and documented ground-glass opacity in the superior segment of the left lower lobe (Figure 2). The patient was hospitalized and received intravenous antibiotic therapy for 12 days with Fosfomycin and Teicoplanin, based on the previous antibiogram that showed colonization with MSSA. This treatment resulted in symptom resolution, improved cough, and an increase in FEV_1_ to 92%. However, at the end of hospitalization, her sputum culture grew *Nocardia* species.

Ten days after completing therapy, she experienced a new episode of fever, cough, and cervical lymphadenopathy with elevated C-reactive protein levels. No new consolidations were identified on the chest X-ray. Nonetheless, the sputum sample confirmed *Nocardia* species. The patient declined further hospitalization and opted for outpatient treatment with oral TMP-SMX and Amoxicillin-Clavulanic acid for 20 days, followed by TMP-SMX alone for 3 months. Subsequently, her symptoms resolved.

A chest CT scan performed two months later showed the resolution of the consolidation, with no progression of bronchiectasis. Lung function tests remained stable and *Nocardia* species have not been detected in sputum cultures to 31 December 2024.

### 2.3. Case 3

We present the case of a 29-year-old woman with bronchiectasis. CF, PCD, and immunological diseases were excluded.

A chest CT scan performed before *Nocardia* detection revealed diffuse cylindrical bronchiectasis in the medial segment of the left lower lobe and, to a lesser extent, in the lingula. Multiple areas of ground-glass opacity and some hyperdense streaks adherent to the parietal pleura were also observed in correspondence with the left anterolateral costophrenic sinus, likely representing disventilatory-fibrotic changes.

She experiences approximately five pulmonary exacerbations per year, necessitating at least one course of intravenous antibiotic therapy. Her median FEV_1_ was 87%. Sputum microbiology demonstrated colonization with *Klebsiella pneumoniae* and *Serratia marcescens*.

At age 26, *Nocardia* was isolated for the first time from a sputum culture during a respiratory exacerbation characterized by chest pain, fever, increased cough, worsening sputum, and a significant decline in spirometry (FEV_1_ decreased from 87% to 73%). Chest CT scan revealed disease progression, with cylindrical bronchiectasis in the medial segment of the left lower lobe and, to a lesser extent, in the lingula, along with subtle consolidative-atelectatic parenchymal changes in the middle lobe, lingula, and both lower lobes. Some hyperdense streaks adherent to the parietal pleura, likely representing disventilatory-fibrotic change, were observed at the left anterolateral costophrenic sinus.

Therefore, the patient was admitted to the hospital and empirically treated with Amikacin and Cefotaxime for 14 days. Pain resolved, cough improved, and a partial improvement in spirometry parameters (FEV_1_ 83%) was observed. One month later, she presented with fever and cough, requiring hospitalization. Considering her allergy to TMP-SMX and Amoxicillin-Clavulanic acid, and based on the susceptibility testing, she was initiated on oral Amikacin and intravenous Linezolid for 14 days, followed by oral Linezolid for another 30 days. She fully recovered with excellent improvement in lung function (FEV_1_ 92%). *Nocardia* was no longer detected in sputum cultures.

### 2.4. Case 4

We present the case of an 18-year-old male adult, diagnosed with PCD at 17 years of age by genetic testing (homozygosis for c.630 dupA; p.(Ala211 Serfs*6) in the gene *RSPH1*) of both the patient and his parents.

Clinical status at the diagnosis showed normal pulmonary function (FEV_1_ 101%), intermittent PA detection in the respiratory cultures, and chest CT characterized by bronchiectasis in the right middle lobe and lingula. The patient required one intravenous treatment per year and an oral antibiotic course every 3 months.

**Figure 3:**
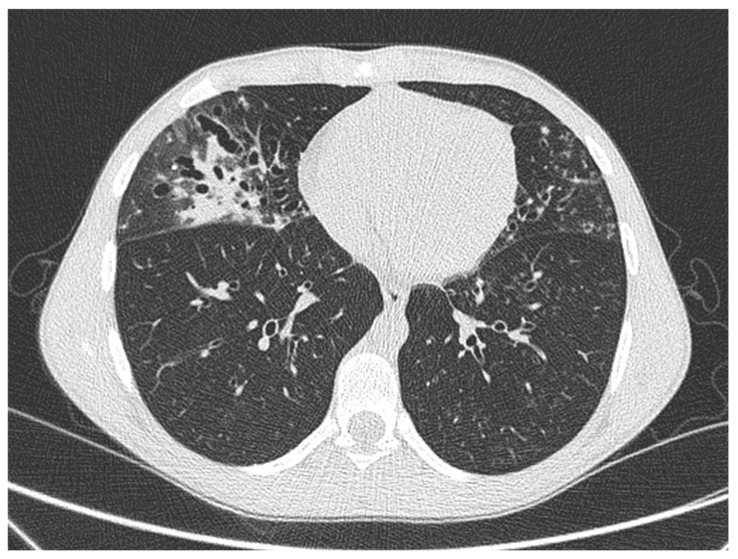
Chest CT in a male young adult affected by PCD, showing bronchiectasis in the middle lobe, with concomitant consolidation and mucous plugs, and new “tree-in-bud” opacities in the lingula.

The patient was admitted to the hospital for a pulmonary exacerbation. Chest CT revealed the progression of bronchiectasis in the middle lobe, with concomitant consolidation and mucous plugs, and new “tree-in-bud” opacities in the lingula, the superior segment of the left lower lobe, and consolidation in the anteromedial part of the left lower lobe (Figure 3). Intravenous treatment with Amikacin, Ceftazidime, and steroids was administered for 14 days, leading to gradual improvement in pulmonary function (FEV_1_ 107%) and the resolution of respiratory symptoms.

After discharge, microbiology testing revealed the isolation of *Nocardia* species. Sputum cultures for nontuberculous mycobacteria were negative and bacterial cultures showed *Haemophilus* spp. Since pulmonary function and clinical status showed significant recovery, no further treatment was prescribed, and close monitoring of cultures and clinical status was conducted.

A new pulmonary exacerbation occurred 7 months later, with middle lobe consolidation on the chest x-ray and a drop in spirometry parameters (FEV_1_ 99%). Treatment with TMP-SMX was then prescribed for 14 days, resulting in clinical, functional and radiological improvement.

After 4 months, the patient presented with a respiratory exacerbation, caused by *Mycoplasma pneumoniae*, and needed intravenous treatment. Microbiological testing was negative for *Nocardia*.

## 3. Discussion

This study focused on the clinical presentation and management of four cases of *Nocardia* isolation in people with CF and non-CF bronchiectasis who experienced clinical and radiological exacerbations attributed to *Nocardia*.

The role of *Nocardia* as a colonizer or pathogen in the respiratory tract remains controversial, as does the optimal treatment strategy. Evidence is scarce and primarily based on case reports and case series, particularly in people with bronchiectasis [7]. A retrospective study [12] suggested that identifying *Nocardia* in sputum cultures does not always indicate active disease but may reflect colonization without associated lung disease. Furthermore, only one case of *Nocardia* has been reported in the literature in patients with PCD, presenting as pleuropneumonia in a 14-year-old girl [13]. In our cohort, all patients presented with a respiratory exacerbation possibly due to Nocardia, suggesting that a prompt therapy could prevent further exacerbation and recurrence.

Diagnosis of *Nocardia* infection can be challenging. *Nocardia* exhibits a slow growth rate, often requiring extended incubation periods for colony formation and susceptibility testing [14]. Additionally, accurate laboratory identification, especially at the species level, can be difficult. In the patients we described, *Nocardia* was isolated from sputum culture. However, alternative sampling methods such as throat swabs/oropharyngeal suction or bronchoalveolar lavage should be considered [15] in suspected cases of Nocardia. Molecular techniques like 16S rRNA gene sequencing can be valuable, particularly in children who are unable to produce sputum or in pwCF receiving ETI treatment [16].

Furthermore, up to 85% of pwCF with *Nocardia* may have a co-infection [7], including *Aspergillus* spp [17,18], *Mycobacterium tuberculosis* [19], and non-tuberculous mycobacteria [20,21]. Therefore, it is crucial to submit samples for stains and cultures for mycetes and mycobacteria. In the reported cohort, a patient had a co-infection with *Aspergillus fumigatus* and *Scedosporium apiospermum*, making the clinical picture even more complex. However, all patients in our cohort had PA co-infection, which has been previously reported as a risk factor for *Nocardia* infection [7].

Since the approval of ETI in pwCF, several studies have demonstrated alterations in the lung microbiome, including increased diversity and richness of bacterial species [22] and a reduction in PA detection or other bacteria that are typical in the disease [23,24,25,26,27,28], although this finding may not be consistent when using 16S rRNA gene amplicon sequencing techniques [29]. Notably, *Nocardia* was not detected in sputum cultures from the patient after the ETI initiation.

Typically, *Nocardia* infection presents with a subacute clinical course, characterized by cough, purulent sputum, and fever, with a duration of symptoms ranging from 1 week to 3 months before reaching the diagnosis [30]. In this study, one patient was asymptomatic at the first *Nocardia* isolation; subsequently he developed a pneumonia with pleural effusion, probably due to *Nocardia,* after failing the eradication therapy.

From a radiological perspective, pulmonary consolidation was the most common finding in our case series, with one case also presenting with pleural effusion. Chest CT typically reveals [31] multifocal lung consolidations with ring enhancement following contrast-administration [32] as the most common presentation. Other findings may include ground-glass opacities, patchy consolidations, masses, mediastinal and hilar lymphadenopathy, and pleural thickening, while cavitations are more frequently observed among immunosuppressed patients [33].

Regarding lung function, the described patients exhibited a decline at the time of *Nocardia* detection, corroborating findings from previous studies of pwCF [33,34,35] and similar to what has been observed in the case of *Stenotrophomonas maltophilia* detection [36]. Given this decline, eradication therapy in asymptomatic patients with bronchiectasis, particularly in cases of lung function decline or radiologic changes [7], may be warranted to prevent recurrence and the further deterioration of lung function.

Another crucial aspect is the selection of the antibiotic for *Nocardia* eradication. Due to the slow-growing nature of the organism, empiric therapy should be initiated promptly upon suspicion, while awaiting the isolation and characterization of the *Nocardia* species, which can exhibit varying antimicrobial susceptibility [14]. An Italian review [14] has shown that *Nocardia farcinica* is the most common subspecies in Italy, followed by *Nocardia abscessus*. These species may be susceptible to Cotrimoxazole, Amikacin, and Linezolid, but can demonstrate resistance to beta-lactam antibiotics. Unfortunately, randomized controlled trials investigating *Nocardia* treatment in patients with bronchiectasis are currently lacking. Guidelines from the Infectious Diseases Community of Practice of the American Society of Transplantation [37] recommend empiric therapy with TMP-SMX for stable *Nocardia* detection or as an alternative if desensitization is unsuccessful or contraindicated. Alternative regimes may include Imipenem/meropenem plus Amikacin or Ceftriaxone or Minocycline or Linezolid, for at least 6–12 months. For critical cases, a three-drug regimen such as Imipenem plus Amikacin, Ceftriaxone, or Linezolid combined with TMP-SMX should be considered while awaiting susceptibility testing [38], followed by a switch to oral therapy with TMP-SMX and/or Minocycline after 3–6 weeks [7]. It is important to note that prolonged Linezolid use can be associated with myelosuppression, particularly thrombocytopenia after 4 weeks, as well as neurotoxicity, limiting its suitability for long-term therapy [38]. A similar approach can be considered for patients with bronchiectasis, although further research is needed to evaluate potential adverse effects and drug interactions. In our study, no patients experienced any side effects.

Furthermore, there is no consensus regarding the optimal duration of the treatment. While previous studies have suggested that prolonged therapy may be more effective in reducing *Nocardia* relapses [39], this has not been consistently confirmed, even in patients receiving lower doses of TMP-SMX for shorter durations [40]. It is important to note that our study included different antibiotic regimes and treatment durations, making it challenging to draw definitive conclusions. However, given the potential for exacerbations probably due to *Nocardia*, it is better to treat with an antibiotic regime which can cover the significant microorganisms identified, including *Nocardia*.

This study focused exclusively on pulmonary manifestations, as none of the patients exhibited dermatological or neurological manifestations. However, some authors recommend brain imaging, such as MRI, in cases of pulmonary nocardiosis, even in the absence of neurological symptoms [41], particularly in immunosuppressed individuals.

The study has limitations inherent to its retrospective design, such as difficulty in identifying *Nocardia* species. Additionally, the small sample size and the patient population heterogeneity, including underlying lung diseases and treatment centers, limit the ability to draw robust comparisons.

**Figure 4:**
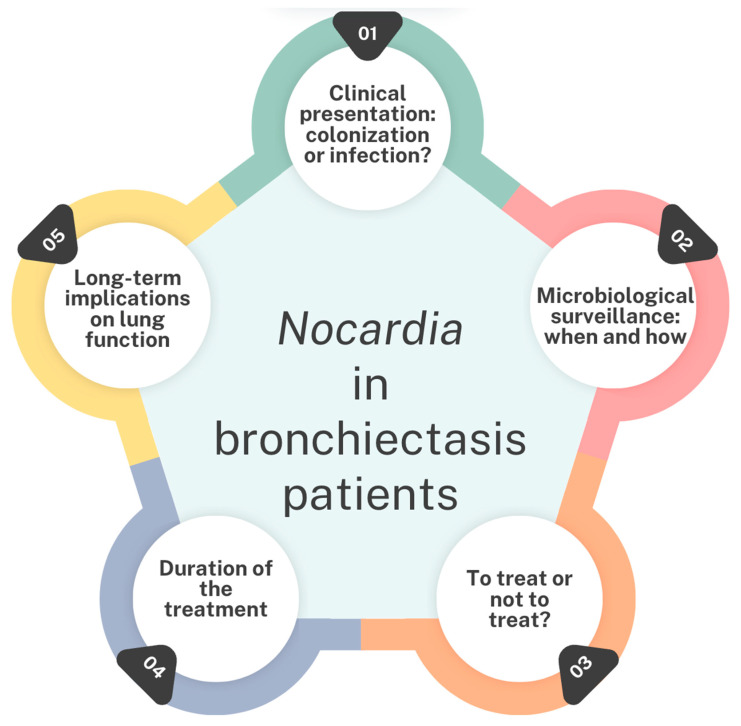
Current gaps in the management of *Nocardia* in bronchiectasis patients [36].

Despite of these limitations, our findings highlight potential concerns regarding the increasing prevalence of *Nocardia* in patients with bronchiectasis. Further research is necessary to address critical knowledge gaps regarding *Nocardia* in both CF and non-CF bronchiectasis populations (Figure 4) [36], including the role of Nocardia in causing respiratory exacerbations, the microbiological surveillance of *Nocardia*, the impact of *Nocardia* detection on lung function and radiological sequelae, and the definition of an appropriate antimicrobial treatment and its duration.

In conclusion, the study highlights the need for larger, multicenter, prospective studies to expand the current findings, helping in detangling these research questions, aiming to ensure optimal care for this uncommon and insidious infection in bronchiectasis patients in the future.

## 4. Materials and Methods

This was a multicenter retrospective cohort study conducted in three Italian centers (Florence, Verona, and Cerignola).

The study was approved by the Ethical Committee of the CF center of Florence on 14 November 2023. Informed consent was obtained from all patients.

Inclusion criteria were a diagnosis of bronchiectasis and a detection of *Nocardia* from cough swab, throat swab, sputum, and broncholavage cultures.

Demographic information, diagnosis, clinical features, and radiological and microbiological reports were collected. Patients were included from 1 January 2014 up to 31 December 2024.

All presumptive *Nocardia* colonies were identified using matrix-assisted laser desorption/ionization time-of-flight mass spectrometry (MALDI-TOF MS Bruker Daltonics Inc., Billerica, MA, USA). However, not all *Nocardia* isolates can be reliably identified beyond the genus level [42].

## Figures and Tables

**Table 1:** Clinical features of the two CF patients with *Nocardia* detections.

Patient ID	Age	Coinfection	Clinical Symptoms	Imaging	FEV1 (%)		BMI (Kg/m^2^)		Treatment and Duration	Eradication
					PRE	POST	PRE	POST		
FI01	33	PA; *A. fumigatus*; SAC	None	Rx: not acute changes in known bronchiectasis	61	61	20.5	20.4	TMP-SMX (30 days)	No
	36	PA; SAC	None	NA	59	65	21.4	20.8	None	No
	37	PA; SAC	More viscous secretions	NA	65	66	20.8	20.9	Ciprofloxacin (14 days)	No
	38	PA; SAC	Fever and cough	Rx: right basal consolidation, with pleural effusion	59	62	20.8	20.5	Ceftazidime and Tobramycin (14 days)	No
	39	PA; *A. fumigatus*; SAC	Fever and cough	Rx: bronchiectasis	63	55	21.6	21.4	TMP-SMX and Ciprofloxacin (14 days)	No
	40	PA	Cough	NA	65	61	21.0	21.0	None	Yes
CE01	26	PA; MSSA	Fever, cough; hemoptysis, lymphadenitis	Rx: left apical consolidation in known bronchiectasis	84	98	19.2	19.5	Amoxicillin-Clavulanic acid and TMP-SMX (14 days)	Yes

Legend: PA, *Pseudomonas aeruginosa;* MSSA, Meticilline sensitive *Staphylococcus aureus;* NA, not available; TMP-SMX, trimethoprim-sulfamethoxazole; SAC, *S. apiospermum* complex.

**Table 2:** Clinical features of the two non-CF bronchiectasis patients with *Nocardia* detections.

Patient ID	Age	Coinfection	Clinical Symptoms	Imaging	FEV1 (%)		BMI (Kg/m^2^)		Treatment and Duration	Eradication
					PRE	POST	PRE	POST		
CE02	26	*Klebsiella pneumoniae; Serratia marcescens*	Fever, cough	Chest CT: consolidation in known bronchiectasis	73	87	23.4	25.1	Amikacin and Cefotaxime (14 days)	Yes
	26	*Klebsiella pneumoniae;* PA	Cough, more viscous secretions	Chest X-rays: not acute changes.	83	92	24.0	23.0	Amikacin and Linezolid (14 days)	
VE01	18	*Haemophilus* spp*.;* PA	Cough, increased secretions	Chest CT: consolidation and mucous plugs, new tree in bud aspect in the lingula, upper and lower lobe, left lower lobe consolidation.	94	107	19.59	NA	Amikacin and Ceftazidime (14 days) ˜	No
			Cough, increased secretions	Chest X-rays: medium lobe consolidation	99	110	19.92	NA	TMP-SMZ (14 days)	Yes

˜ This patient was prescribed steroid therapy. Legend: PA, *Pseudomonas aeruginosa;* NA, not available; TMP-SMZ, trimethoprim-sulfamethoxazole; SAC, *S. apiospermum* complex.

## Data Availability

All the data used for this work are included in the manuscript and available by contacting the corresponding author.

## Abbreviations

The following abbreviations are used in this manuscript:

CF	Cystic fibrosis
CT	Computed tomography
ETI	Elexacaftor/Ttezacaftor/Iivacaftor
FEV_1_	Forced expiratory volume in the first second
MRI	Magnetic Resonance Imaging
MSSA	Meticilline sensitive *Staphylococcus aureus*
NGS	Next-generation sequencing
PA	*Pseudomonas aeruginosa*
PCD	Primary Ciliary Dyskinesia
pwCF	People with cystic fibrosis
SAC	S. *apiospermum* complex
ST	Sweat test
TMP-SMX	Trimethoprim-sulfamethoxazole
